# Evaluation of models for multi-step forecasting of hand, foot and mouth disease using multi-input multi-output: A case study of Chengdu, China

**DOI:** 10.1371/journal.pntd.0011587

**Published:** 2023-09-08

**Authors:** Xiaoran Geng, Yue Ma, Wennian Cai, Yuanyi Zha, Tao Zhang, Huadong Zhang, Changhong Yang, Fei Yin, Tiejun Shui

**Affiliations:** 1 West China School of Public Health and West China Fourth Hospital, Sichuan University, Chengdu, China; 2 Kunming Medical University, Kunming, China; 3 Chongqing Center for Disease Control and Prevention, Chongqing, China; 4 Sichuan Center for Disease Control and Prevention, Chengdu, China; 5 Yunnan Center for Disease Control and Prevention, Kunming, China; Fundacao Oswaldo Cruz, BRAZIL

## Abstract

**Background:**

Hand, foot and mouth disease (HFMD) is a public health concern that threatens the health of children. Accurately forecasting of HFMD cases multiple days ahead and early detection of peaks in the number of cases followed by timely response are essential for HFMD prevention and control. However, many studies mainly predict future one-day incidence, which reduces the flexibility of prevention and control.

**Methods:**

We collected the daily number of HFMD cases among children aged 0–14 years in Chengdu from 2011 to 2017, as well as meteorological and air pollutant data for the same period. The LSTM, Seq2Seq, Seq2Seq-Luong and Seq2Seq-Shih models were used to perform multi-step prediction of HFMD through multi-input multi-output. We evaluated the models in terms of overall prediction performance, the time delay and intensity of detection peaks.

**Results:**

From 2011 to 2017, HFMD in Chengdu showed seasonal trends that were consistent with temperature, air pressure, rainfall, relative humidity, and PM_10_. The Seq2Seq-Shih model achieved the best performance, with RMSE, sMAPE and PCC values of 13.943~22.192, 17.880~27.937, and 0.887~0.705 for the 2-day to 15-day predictions, respectively. Meanwhile, the Seq2Seq-Shih model is able to detect peaks in the next 15 days with a smaller time delay.

**Conclusions:**

The deep learning Seq2Seq-Shih model achieves the best performance in overall and peak prediction, and is applicable to HFMD multi-step prediction based on environmental factors.

## Author summary

Hand, foot and mouth disease (HFMD) remains a serious public health concern in China. It is important to predict trends and understand peaks in the number of cases in advance for its prevention and control. The aim of this study was to consider the influence of meteorology and air pollution on the transmission of HFMD and establish multi-step prediction models for HFMD. In this study, we compared the performance of the Shih attention mechanism with the Luong attention mechanism, Seq2Seq model and LSTM model for future multi-day HFMD prediction based on multi-input multi-output. It was found that the Seq2Seq-Shih model performed best in predicting the trend for the next 2 days-15 days with RMSE, sMAPE and PCC values of 13.943~22.192, 17.880~27.937, and 0.887~0.705, respectively. Meanwhile, it was able to predict the peak within 15 days earlier by 17 days. This is the first study to use Seq2Seq models and perform daily multi-step prediction of HFMD. This study demonstrates the benefit of the Shih attention mechanism in multivariate time series multi-step prediction of infectious diseases.

## 1. Introduction

Hand, foot and mouth disease(HFMD) is a common infectious disease caused by enteroviruses that usually affects children under 5 years [1]. HFMD was first reported in New Zealand [2], and most outbreaks in recent decades have occurred in the Asia-Pacific region [3]. As a country in this region, China listed HFMD as a notifiable infectious disease in 2008 [4]. HFMD was ranked in the top three of all notifiable infectious diseases in China from 2008 to 2019, with an average annual number of approximately 1.87 million cases [5]. According to the study of economic costs associated with patients diagnosed with HFMD in China in 2012–2013, the average quality-adjusted life years (QALYs) lost for mild and severe patients were 6.9 and 13.7 per 1000 cases, respectively. In addition, the average total costs in United States dollars (USD) for mild and severe patients were $1072 and $3051, respectively [6]. In China, HFMD is still a serious public health issue. Therefore, it is necessary to forecast HFMD trends to detect peaks timely, providing more time for public health departments to make decisions and reduce the risk of HFMD outbreaks.

Some environmental factors related to the spread of HFMD need to be considered when forecasting its trends. It has been shown that temperature, humidity, air pressure, rainfall and sunshine duration are associated with HFMD [7–9]. In addition, air pollution factors such as PM_10_, SO_2_ and NO_2_ are also associated with HFMD [10–12]. Furthermore, these environmental factors not only have mostly nonlinear associations with HFMD but also have delayed and cumulative effects on the number of HFMD cases.

In recent years, there have been many studies on the prediction of HFMD [13–23]. For example, Tian et al. developed a seasonal autoregressive integrated moving average (SARIMA) model using monthly data [15]. Xie et al. used the Prophet model to predict daily HFMD cases in Hubei Province, China [19]. Yoshida et al. predicted the weekly number of HFMD cases in Japan [20]. In brief, most of these studies are single time-point predictions, usually predicting the incidence of HFMD on a future day (or week/month). However, for public health departments, the predictions of a single point in time are limited in terms of how far in advance they can detect a peak in the number of HFMD cases. This requires multi-day (week) forecasting to help public health departments to understand the future trends of HFMD cases in advance, to identify peaks early, and to have sufficient time to make decisions. However, there are few studies on the multi-day (week) forecasting of HFMD.

Multi-day (week) forecasting is achieved by multi-step forecasting, i.e., using data from past time points to forecast more than one future time point. There are three main types of multi-step forecasting approaches: direct multi-step [24], iterative multi-step [25], and multi-input multi-output forecasting [26]. Both direct multi-step and iterative multi-step forecasting is achieved by single-step forecasting, which will cause the accumulation of errors and make the forecasting task more difficult. However, multi-input multi-output forecasting is achieved by outputting a prediction vector, which not only avoids error accumulation, but also preserves the random dependences among the numbers of HFMD cases. In addition, considering that meteorological and air pollution factors have delayed and cumulative effects on HFMD and that their effects are nonlinear, we use recursive neural networks to learn these features and achieve multi-day (week) forecasting with multi-input multi-output.

The long-short term memory network (LSTM) model [27] has achieved good performance in predicting infectious diseases such as COVID-19 [28], influenza [29], and dengue fever [30]. It can learn complex nonlinearity, memorize historical information using internal storage units [31], and achieve multi-output through ‘multi-objective regression’.

The sequence-to-sequence(Seq2Seq) model [32] also enables multi-step prediction using LSTM units via an encoder-decoder structure, which converts one sequence (historical cases of HFMD) into another sequence (future cases of HFMD). However, when the length of the input sequence increases, it is more difficult for the encoder to fully encode and store all the information into the context vector, and then the predicted values generated by the decoder are less accurate. The introduction of the attention mechanism into the Seq2Seq model may improve the situation. The attention mechanism learns the most relevant information from all previous data to predict the target sequence [33]. That is, the attention mechanism is able to identify the optimal lag in which the input HFMD, meteorological and air pollution variables have the greatest impact on predicting the number of HFMD cases.

The capital of Sichuan Province, Chengdu is situated in southwest China, west of the Sichuan Basin, and has a humid subtropical monsoon climate. The annual incidence of HFMD in Chengdu showed an increasing trend during 2009–2018, with an average annual incidence of 250.2 per 100,000 person-years [34]. In addition, the major serotypes of HFMD in Chengdu have been changing over the years [35]. Thus, it is extremely important to pay long-term attention to HFMD in Chengdu and to forecast the trend of HFMD for its prevention and control.

In this study, we compared the performance of the Seq2Seq-Shih, Seq2Seq-Luong, LSTM and Seq2Seq models in terms of overall prediction, peak prediction accuracy and prediction time delay by considering the effects of meteorological and air pollution on HFMD in Chengdu city. Seq2Seq-Luong and Seq2Seq-Shih represent the Seq2Seq models using the Luong attention mechanism [36] and the Shih attention mechanism [37], respectively. To our knowledge, this is the first study to use the Seq2Seq model and to make multi-step predictions of HFMD on a daily scale, which can provide public health departments with an advanced understanding of the future trends of HFMD.

## 2. Materials and methods

### 2.1.Ethics statement

Our study was approved by the Institutional Review Board of the West China School of Public Health, Sichuan University. All HFMD surveillance data in this study were obtained from the Chinese Disease Prevention and Control Information System. The study was conducted at the population level. Therefore, this study did not involve confidential information and did not require informed consent.

### 2.2.Data collection and processing

The number of daily HFMD cases in Chengdu from January 1, 2011 to December 31, 2017 was obtained from the Sichuan Center for Disease Control and Prevention. Only cases in patients under 15 years were included in this study since children account for the vast majority of HFMD cases (more than 99% of all cases) [34]. The HFMD surveillance data is not publicly available but it is available on request from the Sichuan Provincial Center for Disease Control and Prevention.

Daily meteorological data were obtained from the site monitoring data of the China Meteorological Data Sharing Service System, including daily average temperature (°C), sunshine duration (h), air pressure (hPa), rainfall (mm), wind speed (m/s) and relative humidity (%). Daily air pollution data were collected from urban monitoring data of the China National Environmental Monitoring Center, including PM_10_ (*μ*g/m^3^), SO_2_ (*μ*g/m^3^) and NO_2_ (*μ*g/m^3^).

First, missing values were addressed. During the study period, there were no missing values for HFMD and meteorological data, 0.16% missing values for SO_2_ and 0.12% missing values for both NO_2_ and PM_10_. We used linear interpolation to fill in these missing values. Next, features were selected. According to the results of Spearman correlation analysis (S1 Fig), air pressure was highly correlated with average temperature, which was excluded from this study because of the stronger correlation between average temperature and HFMD. Next, the data were normalized. To enable the model to handle all features in a balanced way, we normalized each feature to the range [0, 1] using the Min-Max scaling method. Then, the data were divided into 3 parts in chronological order: the training set (80%) was used to build the LSTM, Seq2Seq, Seq2Seq-Luong and Seq2Seq-Shih models, the validation set (10%) was used to adjust the hyperparameters, and the test set (10%) was used to evaluate the overall and peak performance of the models. The overall modeling flow chart is shown in the S2 Fig.

### 2.3.LSTM model

LSTM is a recurrent neural network that is widely used to process temporal data. It stores and controls information flow by constructing internal states and using three gates (input gate, forget gate, and output gate) [38]. The LSTM uses the network activation values of the previous time step as the input of current time step to influence the output of the current time step [39]. This LSTM structure was able to learn the delayed and cumulative effects of influencing factors on HFMD in this study. In addition, LSTM stores network activation values in internal states to learn serial long-term correlations of the number of HFMD cases. Moreover, neural networks are capable of approximating arbitrary nonlinear functions. These properties enable LSTM to model complex multivariate time series [40].

**Fig 1:**
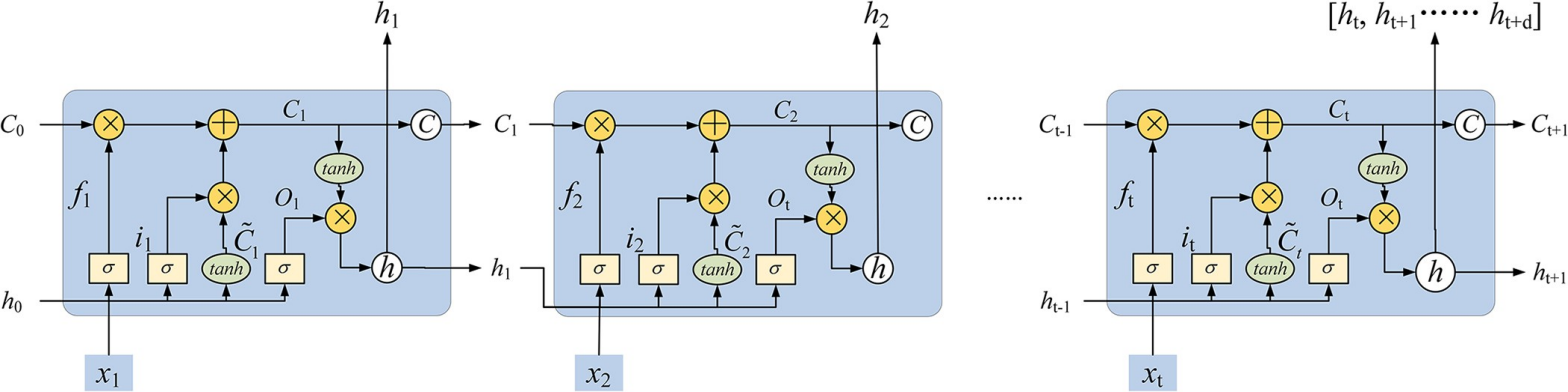
The structure of the LSTM model.

Fig 1 describes the structure of the LSTM model. At each time step, the forget gate (*f*_*t*_), the input gate (*i*_*t*_), the gate of the memory cell (${\overset{˜}{C}}_{t}$), the memory cell (*C*_*t*_), the output gate (*o*_*t*_) and the output (*h*_*t*_) are calculated by the nonlinear function (*σ* or tanh). Here, *h*_*t*−1_, *x*_*t*_, W and b represent the hidden state of the previous time step, the input of the current time step, the weight matrix and the bias, respectively. The last time step outputs row vectors to achieve multi-step prediction.


$$f_{t}=\sigma (W_{f}[h_{t-1},x_{t}]+b_{f})$$ (1)

$$i_{t}=\sigma (W_{i}[h_{t-1},x_{t}]+b_{i})$$ (2)

$${\overset{˜}{C}}_{t}=\tanh\left(W_{c}[h_{t-1},x_{t}]+b_{c}\right)$$ (3)

$$o_{t}=\sigma (W_{o}[h_{t-1},x_{t}]+b_{o})$$ (4)

$$C_{t}=f_{t}\times C_{t-1}+i_{t}\times {\overset{˜}{C}}_{t}$$ (5)

$$h_{t}=o_{t}\times \tanh\left(C_{t}\right)$$ (6)

### 2.4.Seq2Seq model

The Seq2Seq model uses an encoder-decoder structure to convert variable-length input sequences into variable-length output sequences [41], which is originally applied to machine translation and has been increasingly applied to time series prediction in recent years [42]. In our study, the encoder-decoder structure used recurrent neural networks. The encoder learns the information of the input sequence and generates the context vector, while the decoder predicts the output sequence based on the context vector. The LSTM neural unit is the most widely used to learn the long-term dependence of time series. Therefore, the processing unit of both the encoder and decoder in this study used the LSTM unit.

**Fig 2:**
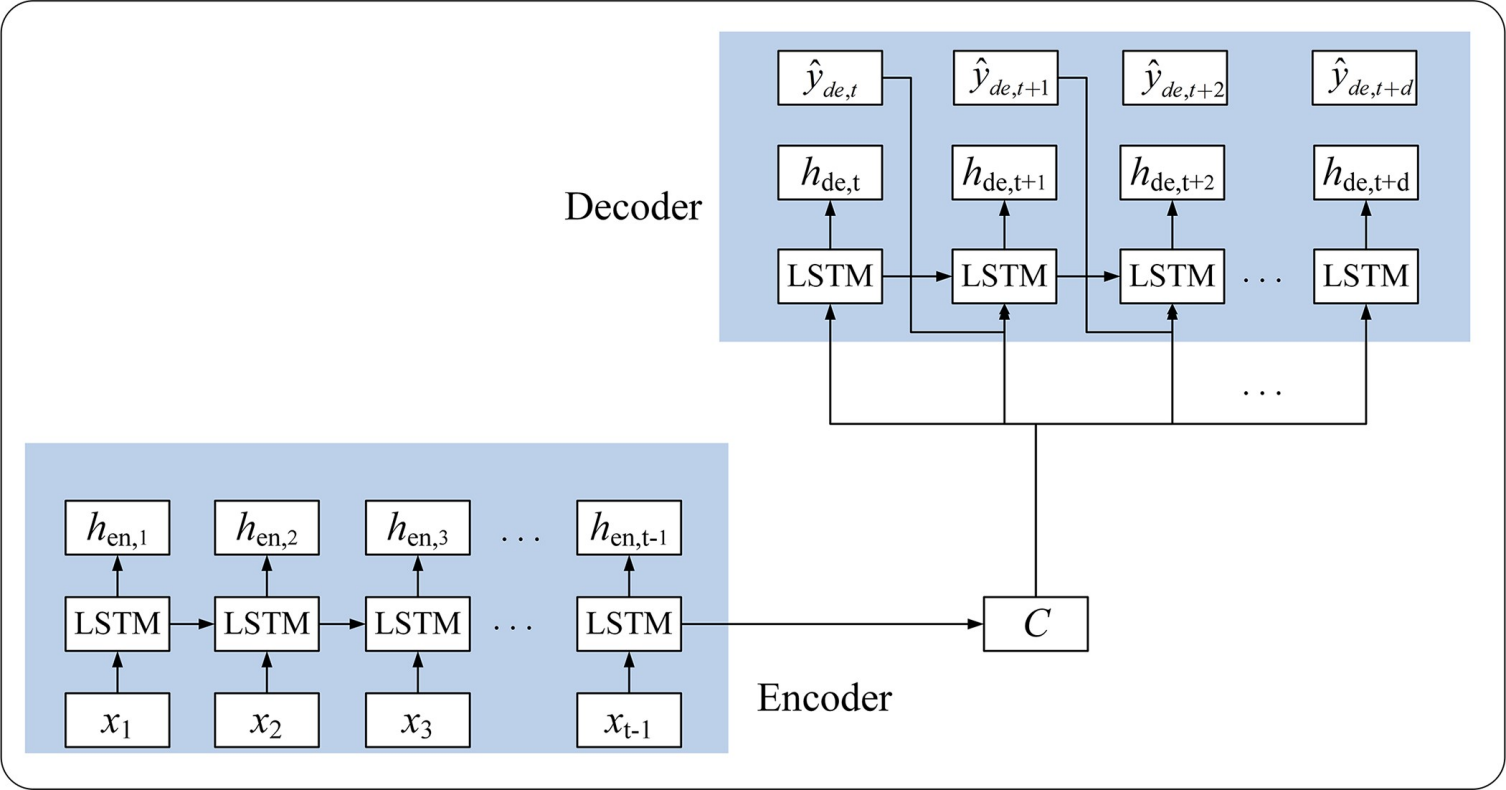
The structure of the Seq2Seq model.

Fig 2 illustrates the structure of the Seq2Seq model. Here, we assumed that ***X***(*x*_1_,*x*_2_,*x*_3_,…*x*_*t*_) represents multivariate time series including the number of HFMD cases and meteorological and air pollution factors at time step *t*, and *y*_*t*_ represents the number of HFMD cases at time step *t*. The encoder generates hidden states *h*_*en*,*t*_ for each time step of the input sequence ***X***. The last time-step hidden state is the context vector *C*. The decoder takes the context vector, the previous hidden state and the previous output as input, calculates the current hidden state output *h*_*de*,*t*+*d*_, and then obtains the current predicted value y^t+d with a nonlinear function. *f*_1_ and *f*_2_ in Eqs (8) and (9) refer to the activation function.


$$\left[h_{en,1},h_{en,2},\ldots h_{en,t}]=\mathrm{LSTM}(X_{1},X_{2},\ldots X_{t}\right)$$ (7)

hde,t+d=f1(y^t+d−1,C,hde,t+d−1) (8)

y^t+d=f2(hde,t+d) (9)

### 2.5.Seq2Seq model based on the Luong attention mechanism

**Fig 3:**
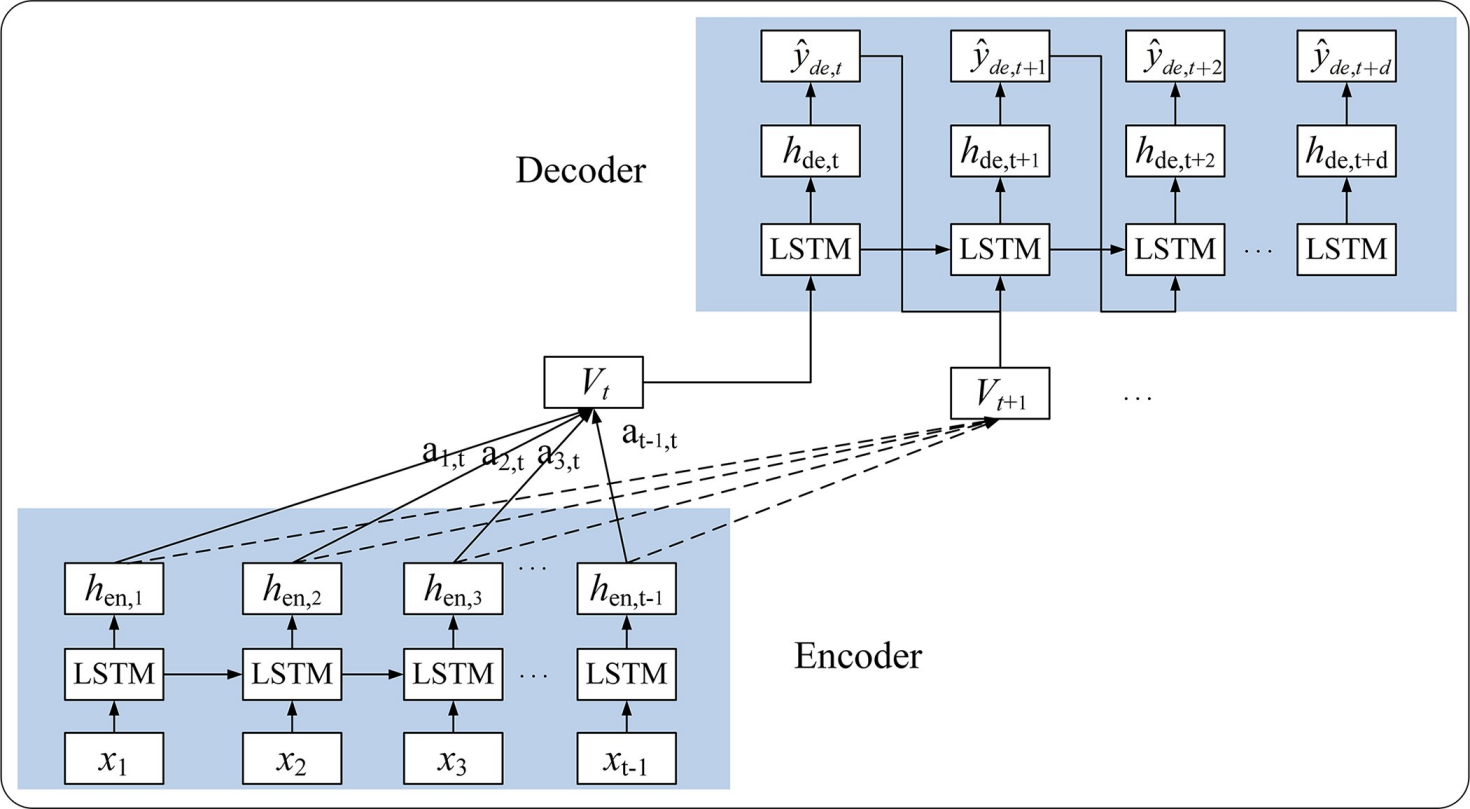
The structure of the Seq2Seq model using the Luong attention mechanism.

The Seq2Seq model makes predictions from the perspective of contextual information in the learning data, and its encoder compresses the contextual information of the input sequence into a fixed-length vector. However, the attention mechanism makes predictions in terms of the relevance of data information, learns the correlations between input and output sequences and focuses on the data features useful for prediction to generate a dynamic contextual vector. The Luong mechanism is proposed for considering all input words in a natural language processing task and the relative importance of each word [36]. Considering the similarity between natural language processing and time series prediction, studies using this method in the fields of energy load [43], wind power [44] and building energy [45] are increasing. The structure of the Seq2Seq model using the Luong attention mechanism is shown in Fig 3.

The context vector *v*_*t*_ in the Luong attention mechanism is calculated by a weighted sum of the hidden states of the encoder and the corresponding attention scores. In this study, we used the ‘dot product’ of the Luong attention mechanism to calculate the attention score. Eq (11) calculates the correlation between the encoder hidden state *h*_*en*,*i*_ and decoder hidden state *h*_*de*,*j*_, which is used to express the importance of *h*_*en*,*i*_ for predicting *h*_*de*,*j*_. The attention score *a*_*ij*_ is aligned using the *softmax* function to highlight the weight of important information.


$$a_{i,j}=\exp\left(score(h_{de,j},h_{en,i})\right)/\sum_{i=1}^{t-1}\exp\left(score(h_{de,j},h_{en,i})\right)$$ (10)

$$score(h_{de,j},h_{en,i})=h_{de,j}^{T}∙h_{en,i}$$ (11)

$$v_{t}=\overset{t-1}{\underset{i=1}{\sum }}a_{i,j}h_{en,i}$$ (12)

### 2.6.Seq2Seq model based on the Shih attention mechanism

**Fig 4:**
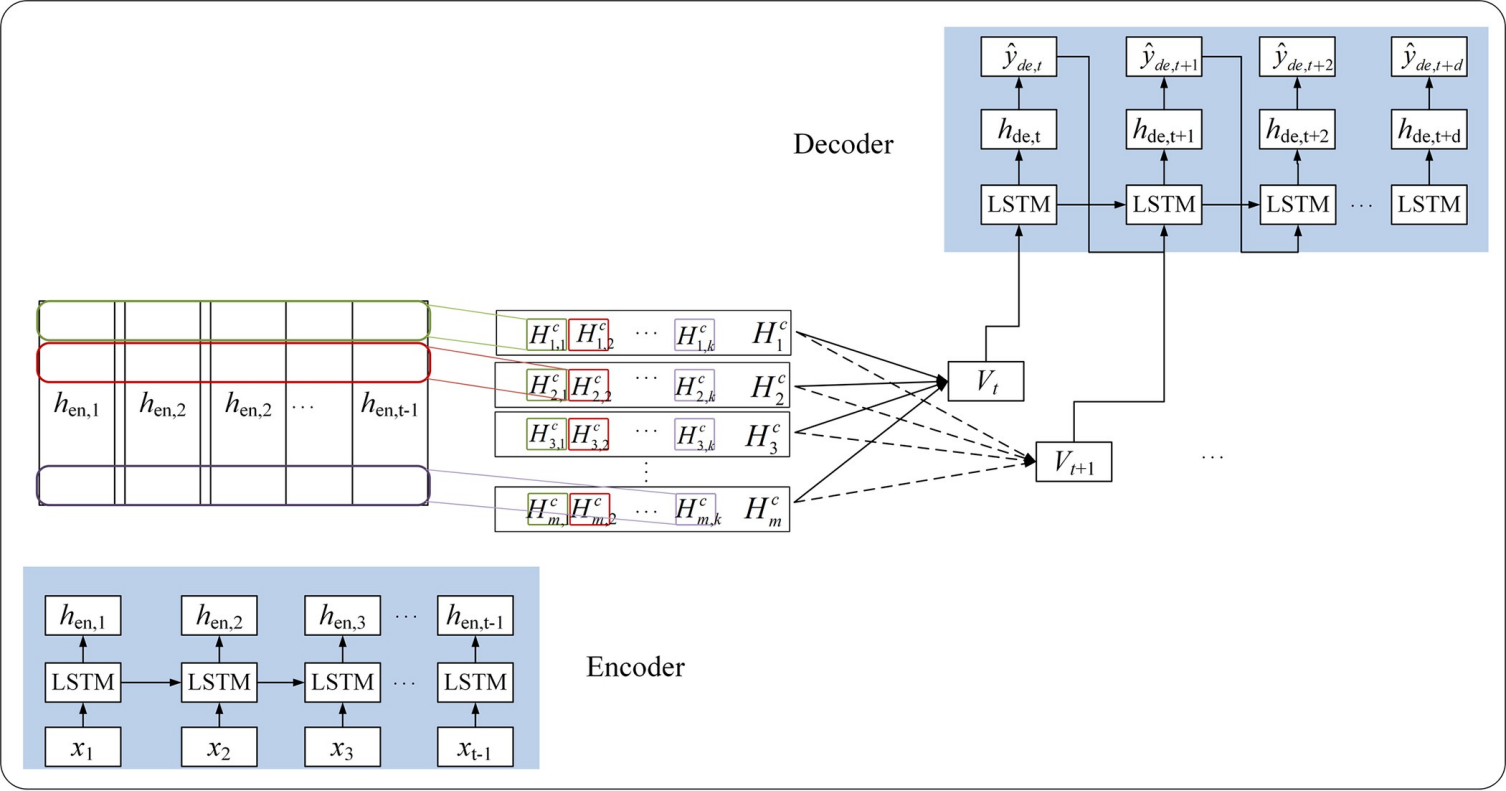
The structure of the Seq2Seq model using the Shih attention mechanism (different colored rectangles indicate 1-D CNN filters).

The Shih attention mechanism focuses on multivariate time series forecasting [31], which selects the relevant variables in each time step on the basis of capturing the time dependence of the time series, while the Luong attention mechanism selects all features of the relevant time step. The encoder of the Seq2Seq model based on these two attention mechanisms is the same, but the decoding process calculates the weights in a different way. The structure of the Seq2Seq model using the Shih attention mechanism is illustrated in Fig 4.

Given the encoder hidden state matrix **H** = $[h_{1},h_{2},\ldots h_{t-1}],\;H\in R^{m\times (t-1)}$, i.e., the encoder extracts the *m*-dimensional information of the data, and the parameters of the LSTM units are shared for each time step; the row vectors of the **H** matrix express the same dimensional information. The Shih attention mechanism uses convolutional neural networks to capture the *m*-dimensional information of the hidden states and produces a matrix ***H***^***C***^. The context vector *v*_*t*_ is obtained by weighting the sum of the attention score and the row vector $H_{i}^{C}$. This method also uses the ‘dot product’ to calculate the attention scores *α*_*i*_ of each row of ***H***^***C***^ with the decoder hidden state *h*_*t*_. *W*_*a*_ represents the connection weights of $H_{i}^{C}$ and *h*_*t*_. Notably, the Luong attention mechanism uses the *softmax* function to scale up the attention of local important information, while the Shih attention mechanism uses the *sigmoid* function to obtain more helpful variables.


$$\alpha_{i}=sigmoid({\left(H_{i}^{C}\right)}^{T}W_{a}h_{t})$$ (13)

$$v_{t}=\overset{m}{\underset{i=1}{\sum }}\alpha_{i}H_{i}^{C}$$ (14)

### 2.7.Parameter selection and model training

**Fig 5:**
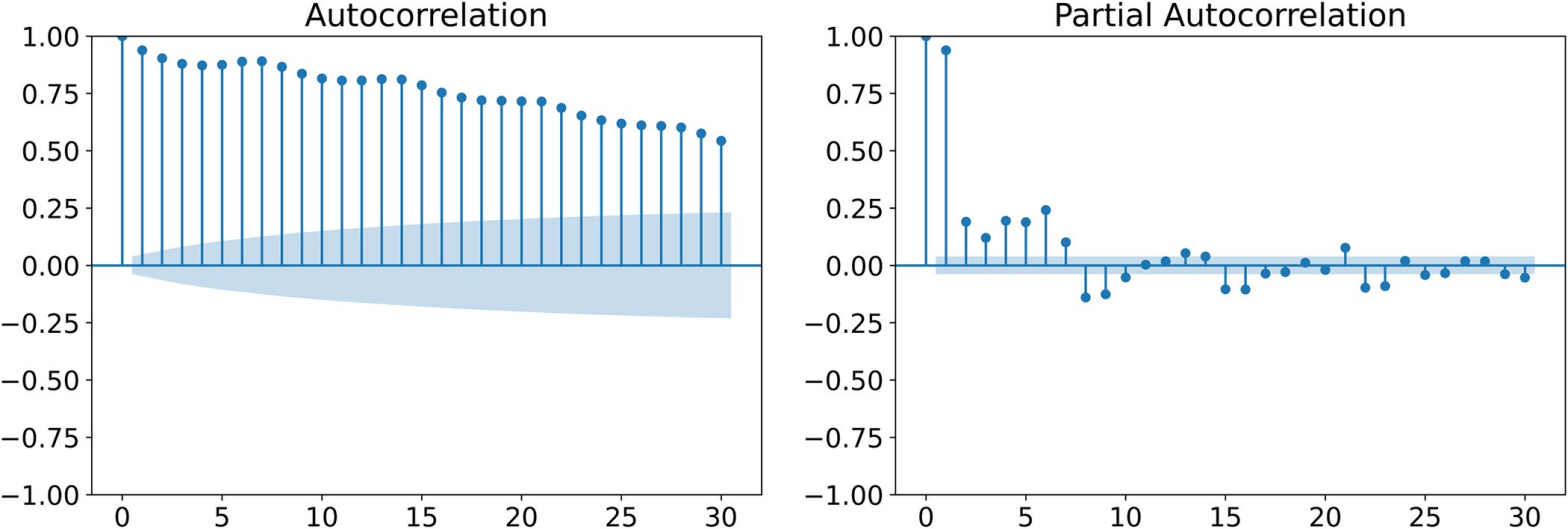
The autocorrelation and partial autocorrelation of HFMD series in Chengdu.

We list some parameters used to train the model. The time step is an important parameter of the neural network. We used the autocorrelation coefficient to calculate the correlation coefficient of the number of HFMD case series at different moments. As seen from Fig 5, the autocorrelation coefficients were tailing and decreasing, and the partial autocorrelation coefficients (PACF) were larger at lags of 1–7 days and then showed a trend with a period of 7 days. However, the trend of the PACF at lags of 1–7 days was different from the PACF during the period, so the time step was set to 14 in this study. The number of hidden layer neurons was searched from the set {16, 32, 64}, the number of encoder layers was obtained from {1, 2}, and the number of decoder layers was set to 1. Moreover, to avoid model overfitting, we also used a dropout parameter to allow neurons to be lost with a 0.2 probability. In the training of models, the loss function was the mean square error (MSE). The number of epochs was determined by the error convergence curve. In addition, considering the randomness of internal parameter initialization during neural network training, we conducted 10 replicate experiments and took the average of the predicted values from the replicate experiments as the final prediction. All models were implemented in Python 3.7 and deployed using Keras 2.6.0 and TensorFlow-gpu 2.6.0.

### 2.8.Model evaluation

#### 2.8.1. Overall performance evaluation

To compare the performance of models, the root mean square error (RMSE), symmetric mean absolute percentage error (sMAPE) and Pearson correlation coefficient (PCC) were calculated.

$$RMSE=\sqrt{\frac{1}{n}\sum_{i=1}^{n}{\left(y_{i}-y_{i}^{*}\right)}^{2}}$$ (15)

$$sMAPE=\frac{2\times 100\%}{n}\sum_{i=1}^{n}\frac{\left|y_{i}^{*}-y_{i}\right|}{\left|y_{i}|+|y_{i}^{*}\right|}$$ (16)

$$PCC=\frac{\sum_{i=1}^{n}\left(y_{i}-{\overset{¯}{y}}_{i}\right)(y_{i}^{*}-{\overset{¯}{y}}_{i}^{*})}{\sqrt{\sum_{i=1}^{n}{\left(y_{i}-{\overset{¯}{y}}_{i}\right)}^{2}}\sqrt{\sum_{i=1}^{n}{\left(y_{i}^{*}-{\overset{¯}{y}}_{i}^{*}\right)}^{2}}}$$ (17)

where *n* represents the length of the observation sequence, $y_{i}^{*}$ is the predicted value of the *i*th observation, *y*_*i*_ is the actual value of the *i*th observation, ${\overset{¯}{y}}_{i}$ is the mean of the observation sequence, and ${\overset{¯}{y}}_{i}^{*}$ is the mean of the predicted sequence.

#### 2.8.2. Peak performance evaluation

We also evaluated the ability of each model to predict the time point and intensity of HFMD peaks. The HFMD peak was defined as the day with the highest number of HFMD cases during the HFMD season. We calculated the difference between the observed peak time *t*_*peak*_ and the predicted peak time t^peak. Moreover, the relative error between the predicted peak value $y_{max}^{*}$ and the observed peak value *y*_*max*_ was also calculated.


delay=|tpeak−t^peak| (18)

$$magnitudeerror=\frac{\left|y_{max}-y_{max}^{*}\right|}{y_{max}}$$ (19)

**Fig 6:**
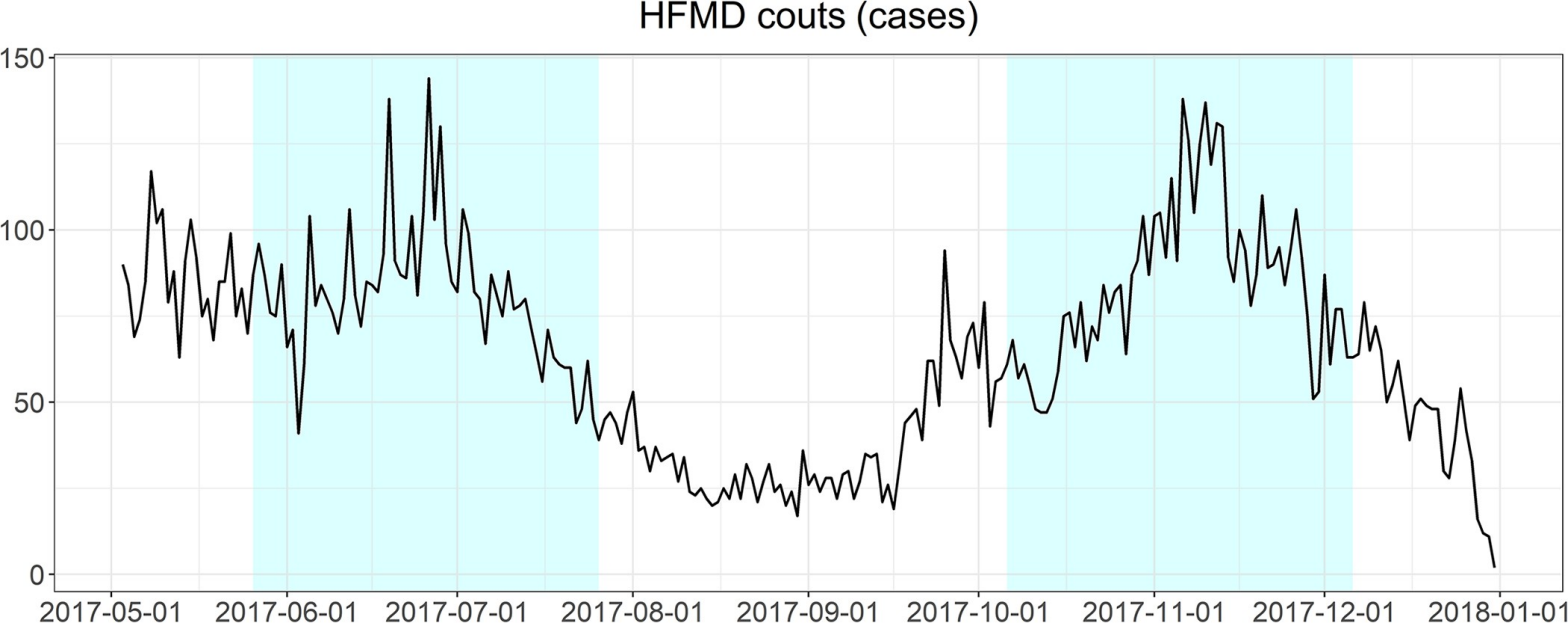
The trends in the number of HFMD cases during the two peak periods in the test set (shaded).

The test set was used to evaluate the performance of the model, so two peak periods were selected from the test set for evaluation in this study. The first peak period was from May 26, 2017 to July 26, 2017 and the second peak period was from October 6, 2017 to December 6, 2017 (Fig 6).

## 3. Results

### 3.1.Descriptive analysis

**Fig 7:**
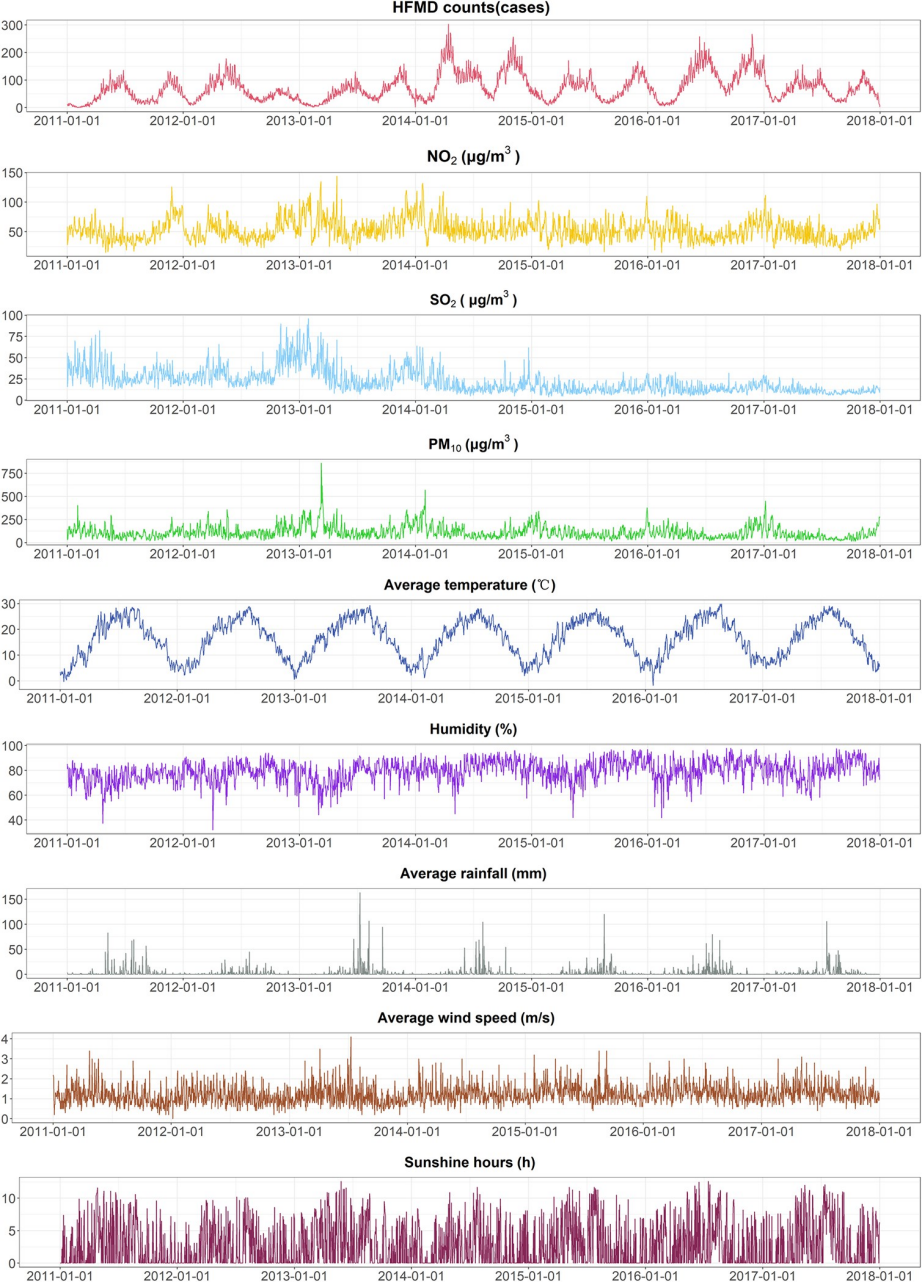
Time series of daily HFMD, meteorological and air pollution variables in Chengdu from 2011 to 2017.

In Chengdu, there were 184,610 HFMD cases in children under 15 years. Approximately 72 HFMD cases were reported daily on average, with a maximum of 303 cases. The average values of wind speed, sunshine duration, temperature, relative humidity, rainfall, PM_10_, SO_2_ and NO_2_ were 1.22 m/s, 2.74 hours, 16.52°C, 79.32%, 2.65 mm, 112.14 *μ*g/m^3^, 21.96 *μ*g/m^3^ and 53.36 *μ*g/m^3^, respectively. Fig 7 shows that there are two annual peaks in the number of HFMD cases, one from April to July and another from October to December. In most years, the first peak is slightly higher than the second, except for 2013 and 2016. Moreover, we observed a clear and consistent seasonal pattern in the number of HFMD cases, temperature, rainfall, relative humidity and PM_10_. The detailed data description of the HFMD, meteorological and air pollution variables are shown in Table 1.

**Table 1:** Descriptive analysis of daily HFMD cases and meteorological and air pollution variables in Chengdu from 2011 to 2017.

Variables	Mean±SD	Min	P_25_	Median	P_75_	Max
HFMD (cases)	72.2±48.46	0	34	64	100	303
wind speed (m/s)	1.22±0.48	0	0.90	1.10	1.50	4.10
sunshine duration (h)	2.74±3.32	0	0	1	5.10	12.60
temperature (°C)	16.52±7.4	-1.80	9.70	17.40	23	29.90
relative humidity (%)	79.32±8.64	32	74.10	80	85.30	98
rainfall (mm)	2.65±9.41	0	0	0	1.20	163.40
PM_10_ (*μ*g/m^3^)	112.14±71.43	15	63	95	141	862
SO_2_ (*μ*g/m^3^)	21.96±13.79	4	12	18	28	96
NO_2_ (*μ*g/m^3^)	53.36±17.98	15	40	50	63	144

Note: SD represents standard deviation, *P*_*x*_ represents the *x*th percentile.

### 3.2.Comparison of models in overall prediction

**Fig 8:**
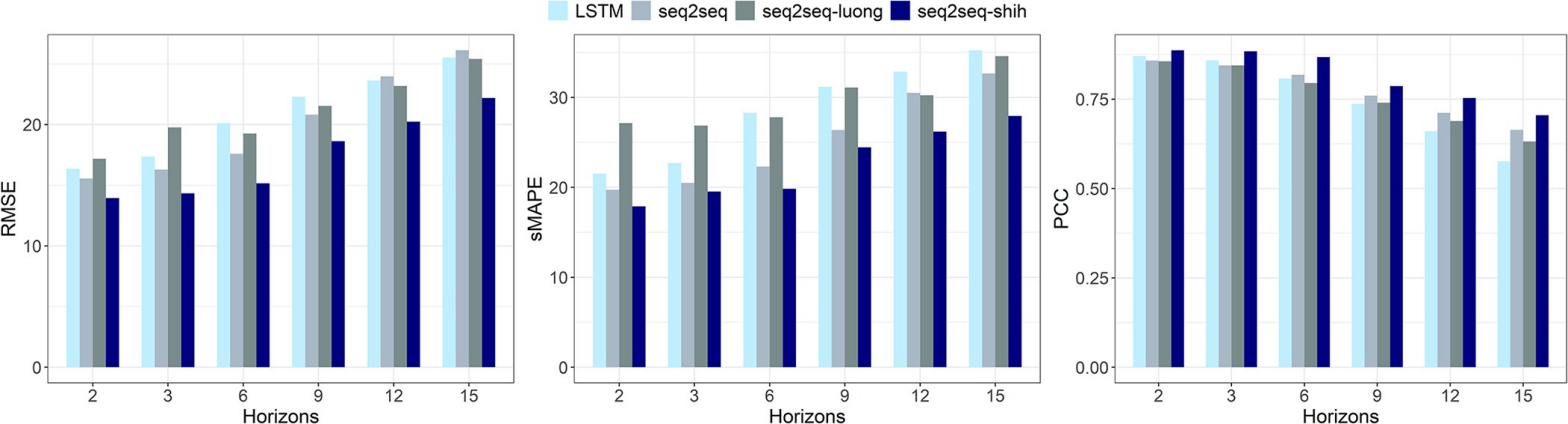
The RMSE, sMAPE and PCC values of models at different forecasting horizons.

**Fig 9:**
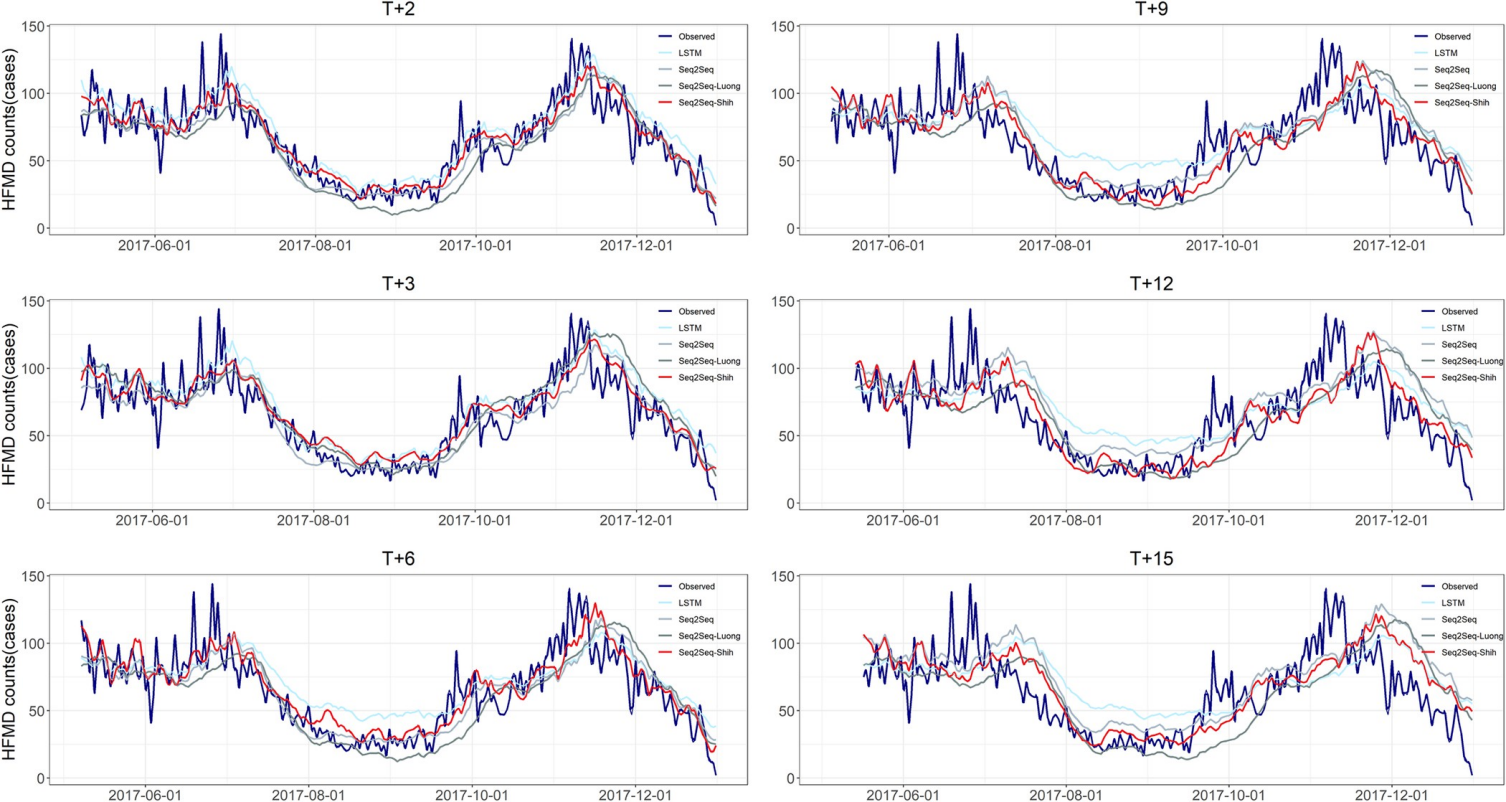
Predictive performance of LSTM, Seq2Seq, Seq2Seq-Luong and Seq2Seq-Shih models for the test set at forecasting steps 2, 3, 6, 9, 12 and 15.

Table 2 summarizes the means and standard deviations of the metrics for the 10 replicate experiments when the model takes the optimal parameters at forecasting steps *h* = 2, 3, 6, 9, 12 and 15, with the bolded numbers indicating the best mean values of the metrics at the forecasting horizons. In addition, we showed the experimental results with bar charts to visually compare the differences of the models (Fig 8). Fig 9 shows the prediction trends of the models at different forecasting horizons and the observed trends.

**Table 2:** Comparison of evaluation metrics of models at different forecasting horizons.

Metrics	Model	Forecasting horizons					
		2	3	6	9	12	15
RMSE	LSTM	16.358 (0.301)	17.361 (0.263)	20.113 (4.915)	22.288 (3.769)	23.635 (1.773)	25.512 (1.053)
	Seq2Seq	15.552 (0.418)	16.290 (0.536)	17.587 (0.700)	20.816 (1.670)	23.971 (1.109)	26.118 (0.837)
	Seq2Seq-Luong	17.182 (1.577)	19.765 (4.282)	19.255 (1.151)	21.539 (1.385)	23.184 (1.922)	25.413 (1.048)
	Seq2Seq-Shih	**13.943** **(1.418)**	**14.337** **(0.900)**	**15.150** **(1.253)**	**18.635** **(0.505)**	**20.239** **(0.539)**	**22.192** **(1.124)**
sMAPE	LSTM	21.522 (0.485)	22.701 (0.451)	28.279 (6.250)	31.162 (5.005)	32.861 (2.856)	35.229 (1.645)
	Seq2Seq	19.719 (1.232)	20.498 (1.233)	22.306 (1.372)	26.372 (2.915)	30.505 (1.847)	32.655 (1.434)
	Seq2Seq-Luong	27.134 (5.813)	26.852 (5.394)	27.783 (5.657)	31.094 (6.489)	30.239 (5.748)	34.585 (2.970)
	Seq2Seq-Shih	**17.880** **(2.291)**	**19.525** **(1.997)**	**19.839** **(2.661)**	**24.445** **(1.622)**	**26.192** **(1.322)**	**27.937** **(1.346)**
PCC	LSTM	0.871 (0.001)	0.859 (0.002)	0.808 (0.010)	0.737 (0.007)	0.661 (0.018)	0.576 (0.014)
	Seq2Seq	0.858 (0.002)	0.844 (0.004)	0.818 (0.008)	0.760 (0.012)	0.712 (0.013)	0.664 (0.018)
	Seq2Seq-Luong	0.855 (0.005)	0.845 (0.010)	0.795 (0.011)	0.740 (0.017)	0.688 (0.029)	0.632 (0.021)
	Seq2Seq-Shih	**0.887** **(0.001)**	**0.884** **(0.002)**	**0.868** **(0.003)**	**0.786** **(0.007)**	**0.753** **(0.014)**	**0.705** **(0.017)**

Note: (*x*) represents the standard deviation of the metrics for 10 replicate experiments.

As shown in Table 2 and Fig 8, the metrics gradually decreased as the forecasting horizon increased, except for the RMSE metric of the Seq2Seq-Luong model at forecasting horizon T+3. In terms of RMSE, the error of the Seq2Seq-Shih model increased from 13.943 at forecasting horizon T+2 to 22.192 at forecasting horizon T+15, with the smallest value for each forecasting horizon and the slowest increase. In terms of sMAPE, the Seq2Seq-Shih model from T+2 to T+15 also obtained the smallest error for each forecasting horizon. In terms of overall consistency between predicted and actual values, the Seq2Seq-Shih model showed better performance for each forecasting horizon, obtaining the largest correlation of 0.887 at forecasting horizon T+2.

Fig 9 indicates that the four models were able to predict the overall trend of HFMD. As the forecasting step increased, the delay in predicting the peak increased and the intensity of the peak was also underestimated. Overall, the prediction curves of the Seq2Seq-Shih model were generally the closest to the observed curves.

### 3.3.Comparison of models in peak prediction

Table 3 shows the average time delay (difference between the predicted and actual peak day) predicted by the model for the two peaks in the test set. At forecasting horizon T+3, the predicted peaks were delayed by 5 days compared to the actual peaks. The Seq2Seq-Shih model had the smallest time delay for all forecasting horizons, with a 2-day delay in predicting the peak for the next two days and a 16.5-day delay in predicting the peak within the next 15 days. The Seq2Seq-Luong model had the largest average time delay for all forecasting horizons.

**Table 3:** Comparison of peak prediction time delay at different forecasting horizons (unit: days).

Model	Forecasting horizons (*h*)					
	2	3	6	9	12	15
LSTM	4	5	8	11	14	17
Seq2Seq	4	5	8	11	14	17
Seq2Seq-Luong	5	5	11.5	14.5	17.5	26
Seq2Seq-Shih	**2**	**5**	**7**	**10**	**13**	**16.5**

Table 4 illustrates the average relative errors of the models in predicting the intensities of the two peaks in the test set. The maximum average relative errors of the LSTM, Seq2Seq, Seq2Seq-Luong and Seq2Seq-Shih models were 0.258, 0.203, 0.271, and 0.207, respectively. The LSTM model had the smallest relative errors at forecasting horizons T+2 and T+3, and the Seq2Seq-Shih model had the second smallest. At forecasting horizon T+6, the peak predicted by the Seq2Seq-Shih model was the closest to the actual peak. The relative error of the peak predicted by the Seq2Seq model was the smallest at forecasting horizons T+9, T+12 and T+15, followed by the Seq2Seq-Shih model. Although the LSTM and Seq2Seq models performed better in peak intensity prediction at some forecasting horizons, they had larger time delays. In addition, they were not as good as the Seq2Seq-Shih model in overall prediction (Fig 9). Therefore, the Seq2Seq-Shih model performed better.

**Table 4:** Comparison of peak prediction error at different forecasting horizons (metrics: magnitude error).

Model	Forecasting horizons (*h*)					
	2	3	6	9	12	15
LSTM	**0.116**	**0.114**	0.238	0.246	0.258	0.252
Seq2Seq	0.193	0.203	0.196	**0.156**	**0.134**	**0.135**
Seq2Seq-Luong	0.264	0.196	0.258	0.251	0.271	0.258
Seq2Seq-Shih	0.187	0.190	**0.151**	0.175	0.164	0.207

## 4. Discussion

In this study, we evaluated the performance of four deep learning models in forecasting future multi-day trends and detecting peaks in the number of HFMD cases in Chengdu. The Seq2Seq-Shih model performed best in overall prediction, and was able to detect a possible peak incidence within half a month, 17 days in advance. This study provides suggestions for multi-day ahead prediction of HFMD to help local health departments respond to upcoming outbreaks in a timely and rapid manner.

The number of infectious disease cases has a strong autocorrelation, with the number of infections at the current moment correlated with the number of recent cases [46]. Therefore, in this study, the time step parameter was determined from the autocorrelation coefficient. The time step parameter was set to 14 for all deep learning models, which is consistent with previous studies on the association between meteorological and air pollution variables and HFMD in Chengdu. There were time lags between environmental factors and the risk of HFMD, with a temperature lag of 0–10 days, a relative humidity lag of 0 day, a wind speed lag of 0–6 days, a PM_10_ lag of 0–14 days, a SO_2_ lag of 0–14 days, and a NO_2_ lag of 0–7 days [11,12,47]. This allowed models to capture the impact of environmental factors on HFMD.

The Seq2Seq model has been used in the time series prediction of thermal load [48], temperature [49,50] and electric vehicle charging demand [51]. In the field of infectious diseases, only malaria [52] and COVID-19 [53] have been studied using this model. Previous studies have found that the Seq2Seq model performs better than the LSTM model [50,52,54]. We also found that the Seq2Seq model outperformed the LSTM model in terms of sMAPE and PCC as well as peak relative error when the forecasting step was greater than 6 days in this study. This may be due to that the Seq2Seq model takes into account the correlation between the number of HFMD cases when making multi-step predictions, making it superior to the LSTM model when making long-term predictions. Surprisingly, the Seq2Seq-Luong model performed poorly in this study, although it showed stronger predictive power than the standalone in other applications [31,45,54]. The advantage of the Luong attention mechanism is that it can learn the importance of the data with different lags in the input sequence for the predicted values; however, this advantage does not seem to be reflected in this study, and regarding this result, we may need more research to determine whether the Seq2Seq-Luong model is truly inferior to the Seq2Seq model in HFMD prediction. In addition, the Seq2Seq-Shih model outperformed the Seq2Seq-Luong and Seq2Seq models, which is consistent with previous findings [31]. This indicates that for the prediction of HFMD, considering the contributions of input variables with different lags to the prediction improves the prediction performance.

Previous studies have used various statistical errors to measure the closeness of the predicted and observed sequences, such as root mean square error (RMSE), mean absolute error (MAE), and mean absolute percentage error (MAPE). These errors provide a general comparison of two sequences. The peak of an epidemic is one of the most important focuses, and knowledge of its magnitude and timing is vital from the perspective of health service providers [55]. The earlier an outbreak is detected, the sooner public health departments can trigger control measures [56]. Therefore, it is also important to assess the performance of peak predictions when making epidemic predictions. For example, Xu et al. used week differences to measure the performance in predicting influenza peaks [57]. Ertem et al. performed model evaluation using peak week error and peak magnitude error metrics [58]. From the perspective of applications, accurate overall forecasting provides epidemiological trends and guides early planning and resource allocation [59], and accurate forecasting of peak times and intensities informs public health departments of changes in the demand for local resources, as well as combines thresholds for outbreak warning [60]. In this study, we evaluated peak predictions using the time delay and magnitude relative error in addition to prediction error and correlation coefficient metrics. The results showed that the Seq2Seq-Shih model had the smallest difference between the predicted peak day and the actual peak day for all forecasting horizons. There was no model consistently had the smallest relative error in peak prediction. However, the Seq2Seq-Shih model remained in the top two performance in peak magnitude prediction. This suggests that the Seq2Seq-Shih model is able to predict the upcoming peak accurately with a smaller time delay.

Previous studies with multi-step prediction of HFMD were conducted at a weekly or monthly scales [14,18]. In terms of model training, integrating daily data into weekly or monthly data results in a smaller sample size of data, which can easily cause the model to be overfitted. Moreover, if meteorological and air pollution data are aggregated into weekly or monthly averages on a daily basis, the number of HFMD cases is inaccurately predicted. In contrast, data with finer temporal resolution reflect trends more accurately and therefore may improve the performance of the model [61]. In addition, daily-scale forecasts of the number of HFMD cases can provide more timely information to facilitate adequate preparation of local medical departments for a possible upcoming HFMD outbreak. Furthermore, our study is the first to predict HFMD trends using the Seq2Seq model, and the results consistently showed that the Seq2Seq-Shih model performed better in overall and peak prediction.

Our study also has some limitations. First, social factors including population flow and population density also influence the spread of HFMD. Further studies are needed to collect and include relevant data in the model. Second, the results obtained in this study were based on Chengdu city. Further studies in other regions are needed to validate the advantages of the Seq2Seq-Shih model for HFMD prediction.

## 5. Conclusions

This study evaluated the performance of four deep learning models in predicting multi-day trends of HFMD based on Chengdu city. The Seq2Seq-Shih model showed high accuracy in predicting future multi-day the number of HFMD cases, along with accurate prediction of peak time and intensity. This study can help public health departments monitor HFMD, understand future HFMD trends and deploy prevention and control measures in advance of an upcoming peak.

## Supporting information

S1 Table Comparison of models used in this study with ARIMA, CNN, XGBoost and random forest models. (DOCX)

S2 Table The hyperparameters adjustment of models in this study at all forecasting horizons (the best parameters are marked in gray for the background). (DOCX)

S3 Table The results of the local sensitivity analysis of models in this study at forecasting horizon T+2. (DOCX)

S1 Fig The Spearman correlation coefficients of HFMD with meteorological and air pollution variables in Chengdu, 2011–2017. (TIF)

S2 Fig The overall modeling flow chart of this study. (TIF)
