# Supplementary material for: Evaluation of models for multi-step forecasting of hand, foot and mouth disease using multi-input multi-output: A case study of Chengdu, China
Source: PLoS Negl Trop Dis. 2023 Sep 8;17(9):e0011587. doi: 10.1371/journal.pntd.0011587 (PMC10511093; doi:10.1371/journal.pntd.0011587)
Supplement: S1 Table — (DOCX) [file pntd.0011587.s001.docx]

**Table S1. Comparison of models used in this study with ARIMA, CNN, XGBoost and random forest models**

| Metrics | Model | Forecasting horizons | | | | | |
| --- | --- | --- | --- | --- | --- | --- | --- |
|  |  | 2 | 3 | 6 | 9 | 12 | 15 |
| RMSE | LSTM | 16.358 | 17.361 | 20.113 | 22.288 | 23.635 | 25.512 |
|  | Seq2Seq | 15.552 | 16.290 | 17.587 | 20.816 | 23.971 | 26.118 |
|  | Seq2Seq-Luong | 17.182 | 19.765 | 19.255 | 21.539 | 23.184 | 25.413 |
|  | Seq2Seq-Shih | **13.943** | **14.337** | **15.150** | **18.635** | **20.239** | **22.192** |
|  | ARIMA | 18.838 | 20.261 | 24.100 | 28.572 | 31.438 | 33.770 |
|  | CNN | 18.132 | 22.077 | 26.002 | 21.352 | 38.125 | 42.505 |
|  | RF | 16.474 | 16.713 | 17.743 | 21.913 | 26.649 | 28.988 |
|  | XGBoost | 16.178 | 15.261 | 16.558 | 21.296 | 23.016 | 29.235 |
| sMAPE | LSTM | 21.522 | 22.701 | 28.279 | 31.162 | 32.861 | 35.229 |
|  | Seq2Seq | 19.719 | 20.498 | 22.306 | 26.372 | 30.505 | 32.655 |
|  | Seq2Seq-Luong | 27.134 | 26.852 | 27.783 | 31.094 | 30.239 | 34.585 |
|  | Seq2Seq-Shih | **17.880** | **19.525** | **19.839** | **24.445** | **26.192** | **27.937** |
|  | ARIMA | 18.270 | 19.720 | 23.438 | 28.442 | 31.665 | 34.201 |
|  | CNN | 24.742 | 29.507 | 32.901 | 35.832 | 45.961 | 49.237 |
|  | RF | 20.102 | 20.848 | 22.344 | 25.848 | 30.366 | 32.773 |
|  | XGBoost | 20.133 | 19.959 | 22.940 | 25.748 | 28.526 | 34.827 |
| PCC | LSTM | 0.871 | 0.859 | 0.808 | 0.737 | 0.661 | 0.576 |
|  | Seq2Seq | 0.858 | 0.844 | 0.818 | 0.760 | 0.712 | 0.664 |
|  | Seq2Seq-Luong | 0.855 | 0.845 | 0.795 | 0.740 | 0.688 | 0.632 |
|  | Seq2Seq-Shih | 0.887 | 0.884 | **0.868** | **0.786** | **0.753** | **0.705** |
|  | ARIMA | **0.904** | **0.890** | 0.845 | 0.781 | 0.738 | 0.697 |
|  | CNN | 0.871 | 0.860 | 0.821 | 0.771 | 0.736 | 0.702 |
|  | RF | 0.852 | 0.856 | 0.845 | 0.782 | 0.716 | 0.677 |
|  | XGBoost | 0.849 | 0.868 | 0.849 | 0.770 | 0.734 | 0.652 |
