# Supplementary material for: Evaluation of models for multi-step forecasting of hand, foot and mouth disease using multi-input multi-output: A case study of Chengdu, China
Source: PLoS Negl Trop Dis. 2023 Sep 8;17(9):e0011587. doi: 10.1371/journal.pntd.0011587 (PMC10511093; doi:10.1371/journal.pntd.0011587)
Supplement: S2 Table — (DOCX) [file pntd.0011587.s002.docx]

**Table S2. The hyperparameters adjustment of models in this study at all forecasting horizons (the best parameters are marked in gray for the background)**

| **model** | **layer_num/encoder_layer** | **units** | **dropout** | **batch_size** | **encoder units** | **decoder units** | **forecasting horizon** | **RMSE** | **sMAPE** | **PCC** |
| --- | --- | --- | --- | --- | --- | --- | --- | --- | --- | --- |
| LSTM | 1 | 64 | / | 32 | / | / | 2 | 16.799 | 21.585 | 0.864 |
| LSTM | 2 | 32 | 0.2 | 32 | / | / | 2 | 17.518 | 21.700 | 0.871 |
| LSTM | 2 | 32 | / | 32 | / | / | 2 | 15.501 | 18.646 | 0.867 |
| LSTM | 2 | 64 | / | 32 | / | / | 2 | 15.978 | 20.069 | 0.863 |
| LSTM | 2 | 64 | 0.2 | 32 | / | / | 2 | 17.469 | 22.898 | 0.862 |
| LSTM | 1 | 64 | / | 32 | / | / | 3 | 19.160 | 25.044 | 0.853 |
| LSTM | 2 | 32 | 0.2 | 32 | / | / | 3 | 21.105 | 26.970 | 0.865 |
| LSTM | 2 | 32 | / | 32 | / | / | 3 | 18.226 | 23.109 | 0.861 |
| LSTM | 2 | 64 | / | 32 | / | / | 3 | 17.665 | 21.442 | 0.858 |
| LSTM | 2 | 64 | 0.2 | 32 | / | / | 3 | 18.704 | 24.249 | 0.853 |
| LSTM | 1 | 64 | / | 32 | / | / | 6 | 24.635 | 32.997 | 0.842 |
| LSTM | 2 | 32 | 0.2 | 32 | / | / | 6 | 22.003 | 28.343 | 0.861 |
| LSTM | 2 | 32 | / | 32 | / | / | 6 | 22.146 | 28.598 | 0.857 |
| LSTM | 2 | 64 | / | 32 | / | / | 6 | 23.659 | 30.877 | 0.857 |
| LSTM | 2 | 64 | 0.2 | 32 | / | / | 6 | 24.324 | 32.599 | 0.843 |
| LSTM | 1 | 64 | / | 32 | / | / | 9 | 29.776 | 38.850 | 0.768 |
| LSTM | 2 | 32 | 0.2 | 32 | / | / | 9 | 25.230 | 32.527 | 0.786 |
| LSTM | 2 | 32 | / | 32 | / | / | 9 | 26.085 | 33.953 | 0.784 |
| LSTM | 2 | 64 | / | 32 | / | / | 9 | 27.332 | 37.135 | 0.780 |
| LSTM | 2 | 64 | 0.2 | 32 | / | / | 9 | 29.798 | 38.881 | 0.769 |
| LSTM | 1 | 64 | / | 32 | / | / | 12 | 30.582 | 40.236 | 0.735 |
| LSTM | 2 | 32 | 0.2 | 32 | / | / | 12 | 27.192 | 35.035 | 0.758 |
| LSTM | 2 | 32 | / | 32 | / | / | 12 | 27.383 | 35.351 | 0.757 |
| LSTM | 2 | 64 | / | 32 | / | / | 12 | 27.240 | 36.792 | 0.761 |
| LSTM | 2 | 64 | 0.2 | 32 | / | / | 12 | 30.465 | 40.157 | 0.734 |
| LSTM | 1 | 64 | / | 32 | / | / | 15 | 30.647 | 40.444 | 0.677 |
| LSTM | 2 | 32 | / | 32 | / | / | 15 | 28.719 | 36.310 | 0.702 |
| LSTM | 2 | 64 | / | 32 | / | / | 15 | 29.186 | 36.708 | 0.703 |
| LSTM | 2 | 64 | 0.2 | 32 | / | / | 15 | 30.549 | 40.324 | 0.673 |
| Seq2Seq | 1 | / | 0.2 | 32 | 32 | 32 | 2 | 17.712 | 21.850 | 0.879 |
| Seq2Seq | 1 | / | 0.2 | 32 | 64 | 32 | 2 | 17.282 | 21.619 | 0.866 |
| Seq2Seq | 1 | / | 0.2 | 32 | 32 | 32 | 3 | 18.789 | 22.982 | 0.875 |
| Seq2Seq | 1 | / | 0.2 | 32 | 64 | 32 | 3 | 16.582 | 19.872 | 0.857 |
| Seq2Seq | 1 | / | 0.2 | 32 | 32 | 32 | 6 | 20.750 | 25.885 | 0.862 |
| Seq2Seq | 1 | / | 0.2 | 32 | 64 | 32 | 6 | 17.312 | 21.482 | 0.821 |
| Seq2Seq | 1 | / | 0.2 | 32 | 32 | 32 | 9 | 19.706 | 23.673 | 0.793 |
| Seq2Seq | 1 | / | 0.2 | 32 | 64 | 32 | 9 | 19.522 | 24.046 | 0.765 |
| Seq2Seq | 1 | / | 0.2 | 16 | 64 | 32 | 9 | 25.556 | 32.667 | 0.789 |
| Seq2Seq | 1 | / | 0.2 | 32 | 32 | 32 | 12 | 20.399 | 24.365 | 0.742 |
| Seq2Seq | 2 | / | 0.1 | 32 | 32 | 64 | 12 | 20.693 | 25.552 | 0.730 |
| Seq2Seq | 1 | / | 0.2 | 32 | 64 | 32 | 12 | 20.691 | 25.067 | 0.728 |
| Seq2Seq | 1 | / | 0.2 | 32 | 32 | 32 | 15 | 21.981 | 26.177 | 0.696 |
| Seq2Seq | 2 | / | 0.2 | 16 | 64 | 32 | 15 | 25.728 | 31.717 | 0.675 |
| Seq2Seq | 2 | / | 0.2 | 16 | 64 | 64 | 15 | 26.502 | 33.628 | 0.674 |
| Seq2Seq | 1 | / | 0.2 | 32 | 64 | 32 | 15 | 22.622 | 27.639 | 0.674 |
| Seq2Seq | 1 | / | 0.2 | 32 | 64 | 64 | 2 | 18.635 | 24.063 | 0.869 |
| Seq2Seq | 1 | / | 0.2 | 32 | 64 | 64 | 3 | 17.999 | 22.327 | 0.858 |
| Seq2Seq | 1 | / | 0.2 | 32 | 64 | 64 | 6 | 17.107 | 20.949 | 0.823 |
| Seq2Seq | 1 | / | 0.2 | 32 | 64 | 64 | 9 | 19.724 | 24.286 | 0.760 |
| Seq2Seq | 1 | / | 0.2 | 32 | 64 | 64 | 12 | 21.358 | 26.028 | 0.712 |
| Seq2Seq | 1 | / | 0.2 | 32 | 64 | 64 | 15 | 23.348 | 28.637 | 0.650 |
| Seq2Seq_Shih | 1 | / | 0.2 | 32 | 64 | 32 | 2 | 22.181 | 30.106 | 0.875 |
| Seq2Seq_Shih | 1 | / | 0.2 | 48 | 64 | 32 | 2 | 20.255 | 27.292 | 0.874 |
| Seq2Seq_Shih | 1 | / | 0.2 | 64 | 64 | 32 | 2 | 14.490 | 18.246 | 0.874 |
| Seq2Seq_Shih | 2 | / | 0.1 | 32 | 16 | 64 | 2 | 20.530 | 26.638 | 0.872 |
| Seq2Seq_Shih | 1 | / | 0.2 | 32 | 64 | 64 | 2 | 23.628 | 31.611 | 0.873 |
| Seq2Seq_Shih | 1 | / | 0.2 | 32 | 64 | 32 | 3 | 24.703 | 32.738 | 0.867 |
| Seq2Seq_Shih | 1 | / | 0.2 | 48 | 64 | 32 | 3 | 20.602 | 27.516 | 0.867 |
| Seq2Seq_Shih | 1 | / | 0.2 | 64 | 64 | 32 | 3 | 15.069 | 19.347 | 0.863 |
| Seq2Seq_Shih | 2 | / | 0.2 | 32 | 32 | 32 | 3 | 21.154 | 28.014 | 0.862 |
| Seq2Seq_Shih | 1 | / | 0.2 | 32 | 64 | 64 | 3 | 26.790 | 35.505 | 0.866 |
| Seq2Seq_Shih | 1 | / | 0.2 | 32 | 64 | 32 | 6 | 25.872 | 33.262 | 0.841 |
| Seq2Seq_Shih | 1 | / | 0.2 | 48 | 64 | 32 | 6 | 20.934 | 26.987 | 0.844 |
| Seq2Seq_Shih | 1 | / | 0.2 | 64 | 64 | 32 | 6 | 16.245 | 20.341 | 0.838 |
| Seq2Seq_Shih | 1 | / | 0.2 | 32 | 64 | 64 | 6 | 27.336 | 33.456 | 0.842 |
| Seq2Seq_Shih | 1 | / | / | 64 | 32 | 32 | 9 | 18.481 | 23.211 | 0.787 |
| Seq2Seq_Shih | 1 | / | 0.2 | 32 | 64 | 32 | 9 | 25.179 | 29.830 | 0.783 |
| Seq2Seq_Shih | 1 | / | 0.2 | 48 | 64 | 32 | 9 | 19.480 | 24.681 | 0.786 |
| Seq2Seq_Shih | 1 | / | 0.2 | 64 | 64 | 32 | 9 | 18.620 | 22.720 | 0.776 |
| Seq2Seq_Shih | 1 | / | 0.2 | 32 | 64 | 64 | 9 | 27.004 | 32.519 | 0.786 |
| Seq2Seq_Shih | 1 | / | 0.2 | 32 | 64 | 32 | 12 | 24.796 | 29.458 | 0.749 |
| Seq2Seq_Shih | 1 | / | 0.2 | 48 | 64 | 32 | 12 | 20.142 | 25.787 | 0.739 |
| Seq2Seq_Shih | 1 | / | 0.2 | 64 | 64 | 32 | 12 | 20.030 | 24.786 | 0.740 |
| Seq2Seq_Shih | 2 | / | 0.2 | 32 | 16 | 16 | 12 | 20.576 | 25.999 | 0.730 |
| Seq2Seq_Shih | 1 | / | 0.2 | 32 | 64 | 64 | 12 | 29.105 | 32.930 | 0.746 |
| Seq2Seq_Shih | 1 | / | 0.2 | 32 | 64 | 32 | 15 | 25.408 | 30.064 | 0.691 |
| Seq2Seq_Shih | 1 | / | 0.2 | 48 | 64 | 32 | 15 | 22.796 | 29.033 | 0.676 |
| Seq2Seq_Shih | 1 | / | 0.2 | 64 | 64 | 32 | 15 | 22.862 | 29.236 | 0.679 |
| Seq2Seq_Shih | 1 | / | 0.2 | 32 | 64 | 64 | 15 | 29.406 | 33.782 | 0.700 |
| Seq2Seq-Luong | 1 | / | / | 64 | 32 | 32 | 2 | 16.260 | 19.589 | 0.865 |
| Seq2Seq-Luong | 1 | / | 0.2 | 32 | 64 | 32 | 2 | 25.180 | 33.841 | 0.867 |
| Seq2Seq-Luong | 1 | / | 0.2 | 48 | 64 | 32 | 2 | 22.794 | 30.654 | 0.866 |
| Seq2Seq-Luong | 1 | / | 0.2 | 64 | 64 | 32 | 2 | 16.861 | 26.439 | 0.857 |
| Seq2Seq-Luong | 1 | / | 0.2 | 32 | 64 | 64 | 2 | 26.053 | 35.273 | 0.863 |
| Seq2Seq-Luong | 1 | / | / | 64 | 32 | 32 | 3 | 16.758 | 19.999 | 0.852 |
| Seq2Seq-Luong | 1 | / | 0.2 | 32 | 64 | 32 | 3 | 26.312 | 35.043 | 0.854 |
| Seq2Seq-Luong | 1 | / | 0.2 | 48 | 64 | 32 | 3 | 24.835 | 32.950 | 0.852 |
| Seq2Seq-Luong | 1 | / | 0.2 | 64 | 64 | 32 | 3 | 17.160 | 24.416 | 0.840 |
| Seq2Seq-Luong | 1 | / | 0.2 | 32 | 64 | 64 | 3 | 26.352 | 35.221 | 0.853 |
| Seq2Seq-Luong | 1 | / | / | 64 | 32 | 32 | 6 | 19.341 | 26.450 | 0.802 |
| Seq2Seq-Luong | 1 | / | 0.2 | 32 | 64 | 32 | 6 | 28.569 | 36.693 | 0.818 |
| Seq2Seq-Luong | 1 | / | 0.2 | 48 | 64 | 32 | 6 | 25.656 | 33.263 | 0.812 |
| Seq2Seq-Luong | 1 | / | 0.2 | 64 | 64 | 32 | 6 | 18.953 | 26.253 | 0.798 |
| Seq2Seq-Luong | 1 | / | 0.2 | 32 | 64 | 64 | 6 | 29.489 | 37.721 | 0.814 |
| Seq2Seq-Luong | 1 | / | 0.2 | 32 | 64 | 32 | 9 | 29.543 | 37.185 | 0.770 |
| Seq2Seq-Luong | 1 | / | 0.2 | 48 | 64 | 32 | 9 | 21.001 | 26.056 | 0.735 |
| Seq2Seq-Luong | 1 | / | 0.2 | 64 | 64 | 32 | 9 | 21.231 | 29.408 | 0.743 |
| Seq2Seq-Luong | 1 | / | 0.2 | 32 | 64 | 64 | 9 | 32.438 | 40.596 | 0.776 |
| Seq2Seq-Luong | 1 | / | 0.2 | 32 | 64 | 32 | 12 | 30.697 | 38.219 | 0.739 |
| Seq2Seq-Luong | 1 | / | 0.2 | 48 | 64 | 32 | 12 | 22.315 | 28.239 | 0.693 |
| Seq2Seq-Luong | 1 | / | 0.2 | 64 | 64 | 32 | 12 | 23.128 | 30.540 | 0.687 |
| Seq2Seq-Luong | 2 | / | 0.2 | 16 | 64 | 32 | 12 | 32.422 | 39.191 | 0.705 |
| Seq2Seq-Luong | 1 | / | 0.2 | 32 | 64 | 64 | 12 | 34.688 | 42.549 | 0.734 |
| Seq2Seq-Luong | 1 | / | 0.2 | 32 | 64 | 32 | 15 | 28.035 | 34.317 | 0.692 |
| Seq2Seq-Luong | 1 | / | 0.2 | 48 | 64 | 32 | 15 | 24.753 | 31.738 | 0.628 |
| Seq2Seq-Luong | 1 | / | 0.2 | 64 | 64 | 32 | 15 | 25.160 | 33.582 | 0.635 |
| Seq2Seq-Luong | 1 | / | 0.2 | 32 | 64 | 64 | 15 | 33.306 | 39.350 | 0.686 |
| Seq2Seq-Luong | 2 | / | 0.1 | 16 | 64 | 16 | 15 | 34.444 | 40.578 | 0.682 |
