# Supplementary material for: Evaluation of models for multi-step forecasting of hand, foot and mouth disease using multi-input multi-output: A case study of Chengdu, China
Source: PLoS Negl Trop Dis. 2023 Sep 8;17(9):e0011587. doi: 10.1371/journal.pntd.0011587 (PMC10511093; doi:10.1371/journal.pntd.0011587)
Supplement: S3 Table — (DOCX) [file pntd.0011587.s003.docx]

**Table S3. The results of the local sensitivity analysis of models in this study at forecasting horizon T+2**

| Variable replaced with white noise | Model | RMSE | sMAPE | PCC |
| --- | --- | --- | --- | --- |
| humidity | LSTM | 17.195 | 22.932 | 0.868 |
|  | Seq2Seq | 17.679 | 22.565 | 0.873 |
|  | Seq2Seq-Luong | 19.233 | 24.914 | 0.859 |
|  | Seq2Seq-Shih | **14.479** | **19.259** | **0.876** |
| T_mean | LSTM | 16.164 | 21.856 | 0.856 |
|  | Seq2Seq | 17.554 | 22.392 | 0.866 |
|  | Seq2Seq-Luong | 16.201 | 19.547 | 0.844 |
|  | Seq2Seq-Shih | **14.784** | **19.476** | **0.866** |
| sunshine duration | LSTM | 18.868 | 25.992 | 0.858 |
|  | Seq2Seq | 16.744 | 21.384 | 0.857 |
|  | Seq2Seq-Luong | 16.168 | **19.844** | 0.847 |
|  | Seq2Seq-Shih | **15.145** | 19.999 | **0.861** |
| wind speed | LSTM | 17.749 | 23.846 | 0.867 |
|  | Seq2Seq | 18.020 | 23.573 | 0.871 |
|  | Seq2Seq-Luong | 16.765 | 22.628 | 0.857 |
|  | Seq2Seq-Shih | **14.550** | **19.64** | **0.875** |
| rainfall | LSTM | 16.004 | 21.628 | 0.868 |
|  | Seq2Seq | 18.857 | 25.077 | 0.872 |
|  | Seq2Seq-Luong | 18.712 | 25.693 | 0.861 |
|  | Seq2Seq-Shih | **15.255** | **21.479** | **0.875** |
| PM_10_ | LSTM | 16.132 | 21.265 | 0.868 |
|  | Seq2Seq | 17.53112 | 22.53453 | 0.871 |
|  | Seq2Seq-Luong | 17.81987 | 23.00473 | 0.861 |
|  | Seq2Seq-Shih | **14.35572** | **19.27765** | **0.877** |
| NO_2_ | LSTM | 15.145 | 21.395 | 0.867 |
|  | Seq2Seq | 16.592 | 21.747 | 0.865 |
|  | Seq2Seq-Luong | 16.132 | 21.112 | 0.858 |
|  | Seq2Seq-Shih | **14.442** | **18.546** | **0.875** |
| SO_2_ | LSTM | 17.912 | 24.438 | 0.862 |
|  | Seq2Seq | 15.275 | **19.031** | 0.863 |
|  | Seq2Seq-Luong | 16.031 | 20.310 | 0.854 |
|  | Seq2Seq-Shih | **14.638** | 20.367 | 0**.874** |
