# Supplementary figures and images for: Evaluation of models for multi-step forecasting of hand, foot and mouth disease using multi-input multi-output: A case study of Chengdu, China
Source: PLoS Negl Trop Dis. 2023 Sep 8;17(9):e0011587. doi: 10.1371/journal.pntd.0011587 (PMC10511093; doi:10.1371/journal.pntd.0011587)

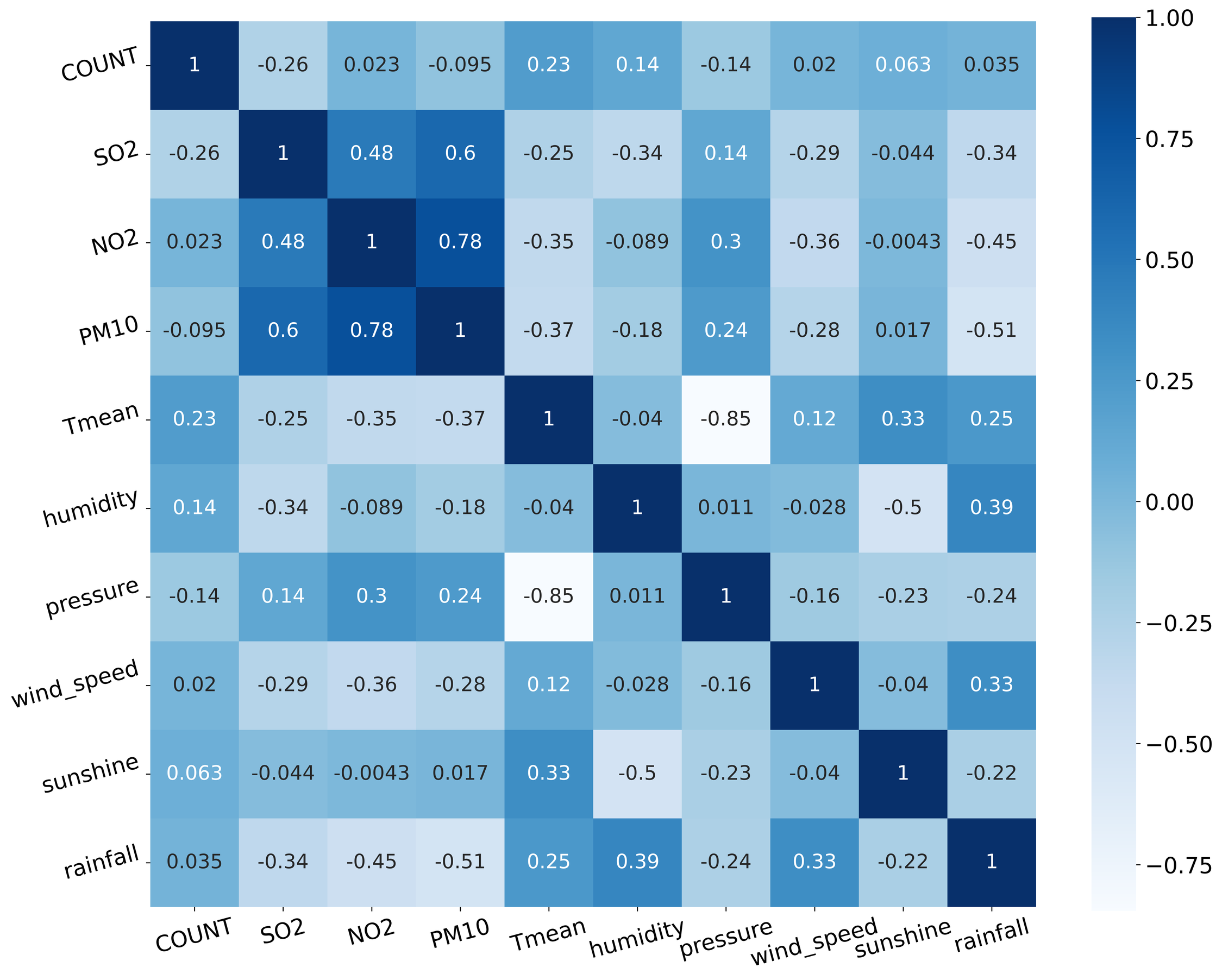

Supplement: S1 Fig — (TIF) [file pntd.0011587.s004.tif]

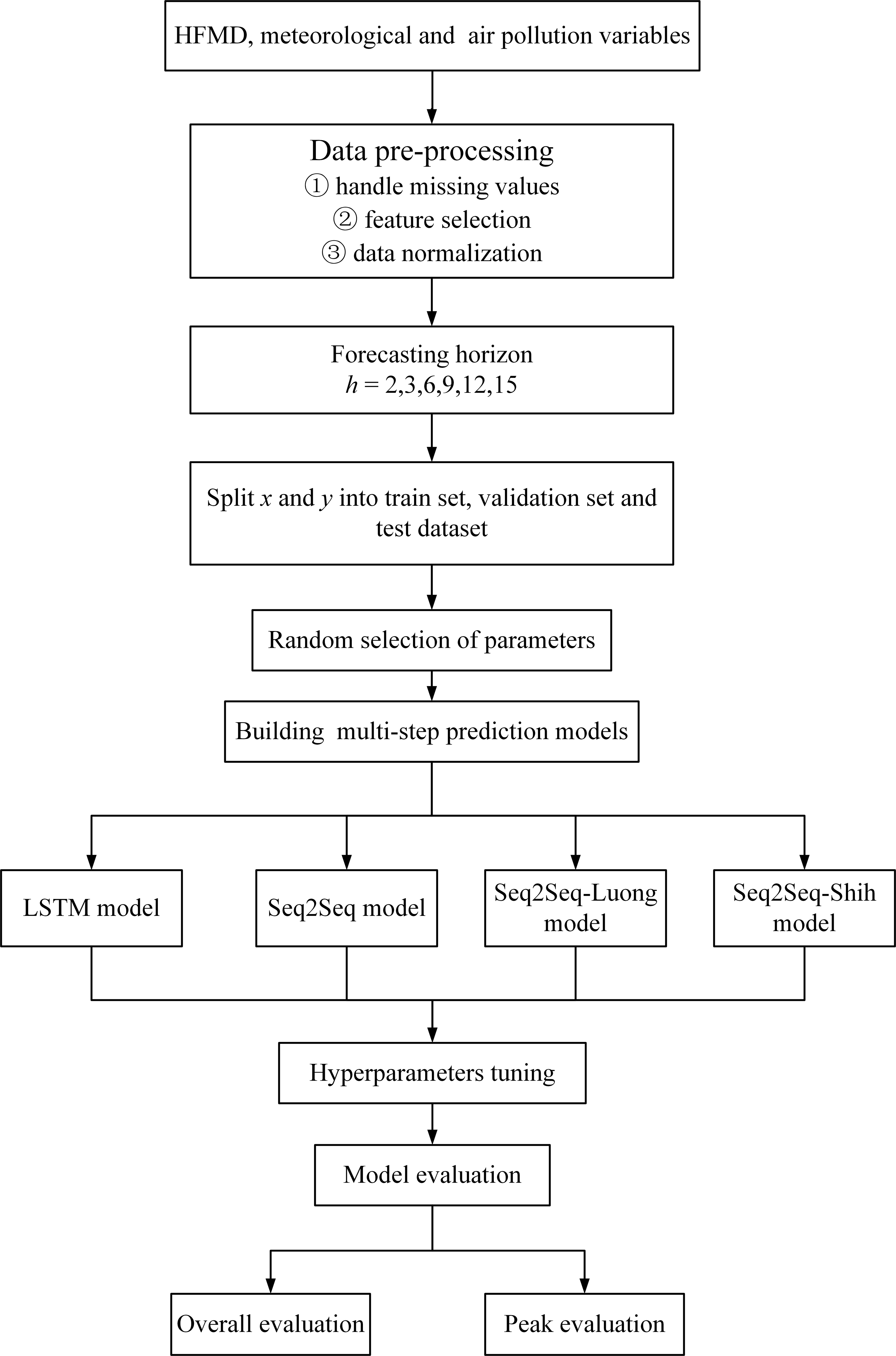

Supplement: S2 Fig — (TIF) [file pntd.0011587.s005.tif]
